# Parkinson’s disease with early motor complications: predicting EQ-5D- 3L utilities from PDQ-39 data in the EARLYSTIM trial

**DOI:** 10.1186/s12955-020-01299-y

**Published:** 2020-03-02

**Authors:** Mehdi Zahra, Isabelle Durand-Zaleski, Michal Górecki, Silke Walleser Autiero, Gillian Barnett, W. M. Michael Schüpbach

**Affiliations:** 1 Medtronic International Trading Sarl, Route de Molliau 31, 1131, Tolochenaz, Tolochenaz, Switzerland; 2 Hôpital de l’Hotel-Dieu, Ile de France, 1 Place du Parvis de Notre-Dame, 75004 Paris, France; 3 Santé Publique Hôpital Henri-Mondor, 51, avenue du Mal de Lattre de Tassigny, 94010 Créteil, France; 4 Health Technology Assessment Consulting, Starowiślna 17/3, 31-038 Kraków, Poland; 5 Gillian Barnett and Associates Limited, Claggan, Hornhead, Dunfanaghy, Letterkenny, Co. Donegal, Dunfanaghy, Ireland; 6 Assistance Publique Hôpitaux de Paris, Institut National de Santé et en Recherche Médicale, Institut du Cerveau et de la Moelle Epinière, Centre d’Investigation Clinique 1422, Département de Neurologie, Hôpital Pitié-Salpêtrière, F-75013 Paris, France; 7 Movement Disorders Center, Department of Neurology, Bern University Hospital and University of Bern, Freiburgstrasse 8, 3010 Bern, Switzerland

**Keywords:** Utility, Quality of life, Parkinson’s disease, Deep brain stimulation

## Abstract

**Background:**

A utility value is a health-related quality of life metric (HRQoL) metric used in a cost-effectiveness analysis. While utilities as outcomes in the treatment of advanced Parkinson’s disease (PD) with deep brain stimulation (DBS) are available, they do not currently exist for PD with early motor complications. The objectives of this study were to predict utilities from observed disease-specific HRQoL data using two mapping algorithms, and investigate their performance in terms of longitudinal changes within and between treatment groups, and distribution by PD severity.

**Methods:**

This is a post hoc analysis of data from the EARLYSTIM trial of DBS compared with best medical therapy (BMT) in PD patients with early motor complications We used two published algorithms comprising ordinal and multinomial regression models to map EQ-5D-3L utilities from observed PD-specific 39 item Questionnaire (PDQ-39) scores in EARLYSTIM. Utilities were calculated using the predicted functioning levels of EQ-5D-3L dimensions and the established EQ-5D-3L UK tariffs. Statistical analyses (analysis of variance, two-tailed Student’s t-test) were used to test the change from baseline within groups and difference in change from baseline between groups in utilities. Boxplots were developed to investigate the distribution of predicted utilities by PD severity, measured using the Hoehn and Yahr scale.

**Results:**

The change from baseline in predicted mean utilities was statistically significant at all visits up to 24 months for the DBS group (*p* < 0.001) with both algorithms, and statistically significant at 12 months only (*p* = 0.04) for the BMT group with one algorithm. With both algorithms, the between-groups difference in change from baseline in predicted mean utilities favored DBS at all follow-up visits (p < 0.001). Based on the Hoehn and Yahr scale, predicted utilities deteriorated with increasing disease severity.

**Conclusions:**

Among PD patients with early motor complications, utilities predicted by both mapping algorithms using PDQ-39 data demonstrated a statistically and clinically meaningful improvement with DBS compared with BMT. It was not possible to conclude if one algorithm was more responsive than other. In the absence of utilities collected directly from patients, mapping is an acceptable option permitting economic evaluations to be undertaken.

## Background

Recognition of the multidimensional impact of Parkinson’s disease (PD) on patients’ health-related quality of life (HRQoL) led to the development of the PD-specific 39 item Questionnaire (PDQ-39) [1], the most commonly used HRQoL instrument in clinical studies in PD [2], with superior clinimetric and psychometric properties compared with other PD-specific instruments [3].

The results of health economic evaluations, most commonly cost-utility analyses, facilitate the efficient allocation of healthcare resources, and ultimately determine clinical practice. Cost-utility analyses require estimates of health-state preferences based on patient ratings, referred to as utilities. Utilities represent the strength of an individual’s preference for specific health-related outcomes (“preference weights”) and are used for calculating quality–adjusted life years, a combination of survival and utilities generated by healthcare interventions, the metric used in cost-utility analyses.

The Euroqol (EQ-5D) is the most widely used and preferred method for generating utilities [4, 5]. The EQ-5D-3L (3 L represents three levels of responses) is a valid tool for measuring utilities in PD as it correlates strongly with PDQ-39 scores and disease severity [6]. In patients with advanced PD implanted with DBS, two studies have reported a positive impact of DBS on utilities, as measured using the EQ-5D-3L [7, 8]. However, to our knowledge, utilities for patients with PD and early motor complications have, thus far, not been reported.

In the absence of utility measurement directly from patients during a clinical trial, an accepted alternative is to derive utilities from observed disease-specific HRQoL instrument scores using mapping algorithms [9]. Two types of mapping algorithms have been developed using different statistical models: Direct utility mapping to directly predict the EQ-5D-3L summary index utility, and response (or indirect) mapping to predict responses to each EQ-5D-3L dimension [10]. To date, only two additional reports describing mapping algorithms in PD have been published [11, 12]. However, as they present algorithms for the estimation of the EQ-5D utilities from the PDQ-8 and the Unified Parkinson’s disease rating scale parts II-IV, neither were suitable for application in the current study.

The objectives of this study were to apply two mapping algorithms to predict utilities from observed disease specific HRQoL data collected in the EARYSTIM trial, and to investigate the performance of the algorithms in terms of change from baseline within groups and difference in change from baseline between groups, and the distribution of the predicted utilities by PD severity.

## Methods

### Study design

A post hoc study was carried out to predict EQ-5D (three level version) (EQ-5D-3L) utilities from observed PDQ-39 subscale scores from the EARLYSTIM trial by applying two peer-reviewed mapping algorithms [13, 14] to EARLYSTIM patient data at baseline, five, 12 and 24 months. To convert EQ-5D-3L scores into utilities from a UK perspective, tariffs specific to the UK were utilized [15] (French tariffs [16] were applied as an additional analysis).

### Instruments

The PDQ-39 consists of 39 items grouped into eight subscales (mobility, activities of daily living (ADL), emotional well-being, stigma, social support, cognitions, communication, and bodily discomfort) [1]. Response levels “never”, “occasionally”, “sometimes”, “often” and “always” are allocated a value from 0 to 4, and then scored to provide a 0–100 index for each subscale and the overall instrument [17]. A higher score indicates a worse HRQoL.

Both Young et al. and Kent et al. utilized the EQ-5D-3L utility index which comprises five dimensions (mobility, self-care, usual activities, pain/discomfort, anxiety/depression). Each dimension has three response levels, “no problems”, “some problems”, or “extreme problems”. A single summary index utility is calculated by attaching weights to the level in each dimension, based on health state valuations derived from the general population. A value of 1.0 represents full health and 0.0 represents death.

The Hoehn and Yahr scale (H&Y) is used to assess the severity of PD in terms of clinical features and functional ability [18, 19]. It’s scale ranges from zero, indicating no disability or impairment, to five, indicating the most advanced disease stage.

### Patient data

Raw data were obtained from the EARLYSTIM study (ClinicalTrials.gov Identifier: NCT00354133) designed to compare subthalamic DBS plus BMT to BMT alone for up to 24 months [20]. To detect a standardized effect size of 0.4 with an alpha level of 5% for the summary index of the PDQ-39, the primary outcome in EARLYSTIM, and assuming a 15% dropout rate, it was estimated that at least 246 patients would be required. Among 392 patients assessed for eligibility, 251 were enrolled at nine German and eight French university centers. The intention-to-treat (ITT) population comprised 124 patients assigned to DBS (120 underwent implantation and completed the study) and 127 patients assigned to BMT (125 underwent BMT and 123 completed the study). The per-protocol analysis included 116 patients in the DBS group and 110 in the BMT group. Included were adults with PD for at least 4 years, aged up to 60 years (mean age 52 years) who reported motor complications for 3 years or less (mean years post onset of 1.7), and scored less than three on the H&Y staging scale while on medication. In contrast to other controlled studies, the EARLYTIM population is unique as evidenced by their young age, short disease duration, mild symptom severity, and short duration of complications [20]. Trial participants completed the PDQ-39 during interviews at baseline, five, 12, and 24 month visits.

In the EARLYSTIM ITT population, the change from baseline through 24 months in the PDQ-39 summary index favored DBS compared with BMT (mean between-group difference in change from baseline, 8.0 ± 1.6 (4.2 to 11.9; *p* = 0.002)). Per protocol results were similar [20].

### Algorithm description

In the absence of EQ-5D-3L utilities for the patient population of interest, two algorithms [13, 14] (Table 1) were applied to longitudinal patient data from EARLYSTIM to estimate EQ-5D utilities from PDQ-39 summary index scores.

**Table 1:** Algorithms used to predict ED-5D summary index utilities

Young et al. [13]	Kent et al. [14]
prob(“no problems”) = *ϑ*_*1*_ = 0.5 – tan^− 1^ (−*α*_*1*_ + *β’X*)/ *π* prob.(“some problems”) = *ϑ*_*2*_ = 0.5 – tan^− 1^ (−*α*_2_ + *β’X*)/ *π*- *ϑ*_*1*_ prob.(“extreme problems”) = *ϑ*_*3*_ = 1 – *ϑ*_*1*_ – *ϑ*_*2*_ *Where α*_*i*_ (*i*_=_ 1; 2) were the constant terms in the linear predictor for “no problems” and “some problems,” respectively, and *β’X* in the linear predictor related the functioning levels of an EQ-5D-3L dimension with the PDQ-39 subscale scores. Based on individual pattern X, the predicted functioning level can be obtained by assigning the category with the largest estimated probability (that is, the maximum of *ϑ*_*1,*_*ϑ*_*2, and*_*ϑ*_*3*_)	Dimension i = β(Dimension, i)*x Where i = 1,2,3. Dimension i corresponds to getting a response to an EQ-5D-3L question (i.e. Mobility 2 indicates the response “some problems” in the Mobility dimension). β is the vector of the regression coefficients. x is the matrix of the PDQ-39 scores of the subscales. $P\ \left( Dimension\ i\right)=\frac{e^{Dimension\ i}}{e^{Dimension\ 1}+{e}^{Domension\ 2}+{e}^{Dimension\ 3}}$ Where i = 1,2,3, and Dimension 1 = 0 (The pivot outcome); e^x^ is the natural exponential function

The algorithm developed by Young et al. [13] comprises an ordinal regression model with the Cauchit (inverse Cauchy) link developed by a direct utility mapping model. This mapping model used data from a sample of community-dwelling adults in Australia with advanced PD (*N* = 96) who had been administered the PDQ-39 and the EQ-5D. The second algorithm is a multinomial regression model with logit link function developed by Kent et al. [14], a response mapping model with age and gender as additional covariates, which was developed by applying pairs of complete responses to the PDQ-39 and the EQ-5D from the UK PD MED trial [21].

### Data analysis

Baseline and five, 12- and 24-month follow-up PDQ-39 subscale scores for the DBS group and the BMT group in the EARLYSTIM trial provided a common data set for application of the best performing mapping models, the ordinal regression model with Cauchit link function [13], and the multinomial regression model with logit link function (controlling for age and gender) [14].

Based on the regression coefficients derived from the ordinal and multinomial regression models, the ensuing equations with three predicted functioning (response) levels for each of the five EQ-5D-3L dimensions were applied to the longitudinal PDQ-39 data of EARLYSTIM patients to predict EQ-5D-3L utilities. The EQ-5D-3L summary index utilities were therefore calculated using the predicted functioning levels of EQ-5D-3L dimensions and the established EQ-5D-3L tariffs for the UK [15]. The EQ-5D-3L tariffs for France [16] were applied as an additional analysis.

As the number of missing data cases was low (four BMT cases out of 127 (3%), and five DBS cases out 124 (4%)), a complete cases approach was applied.

Repeated measure analysis (analysis of variance) was used to test the between treatment differences in utilities over time. The change from baseline within treatment groups and the differences in the mean change from baseline between treatment groups were analysed at each follow-up visit using the two-tailed Student’s t-test at a significance level of 95%. A multiple test correction (Bonferroni correction) was performed to adjust the *p*-values and confidence intervals for multiple testing (setting the level of significance to 0.05/3(0.017) when performing three tests).

To explore the face validity of the predicted EQ-5D-3L utilities, the relationships between both the predicted EQ-5D-3L utilities and observed PDQ-39 scores and the H&Y scale were investigated. Mean summary PDQ-39 scores by mean H&Y stage were compared to the predicted mean EQ-5D-3L utilities by mean H&Y stage. Boxplots of both measures were generated to explore the variability of predicted EQ-5D-3L and observed PDQ-39 values by H&Y stage. Observations from all visits throughout EARLYSTIM were used to assess this relationship for each H&Y stage.

All calculations were conducted in R Statistical Software.

## Results

**Fig. 1:**
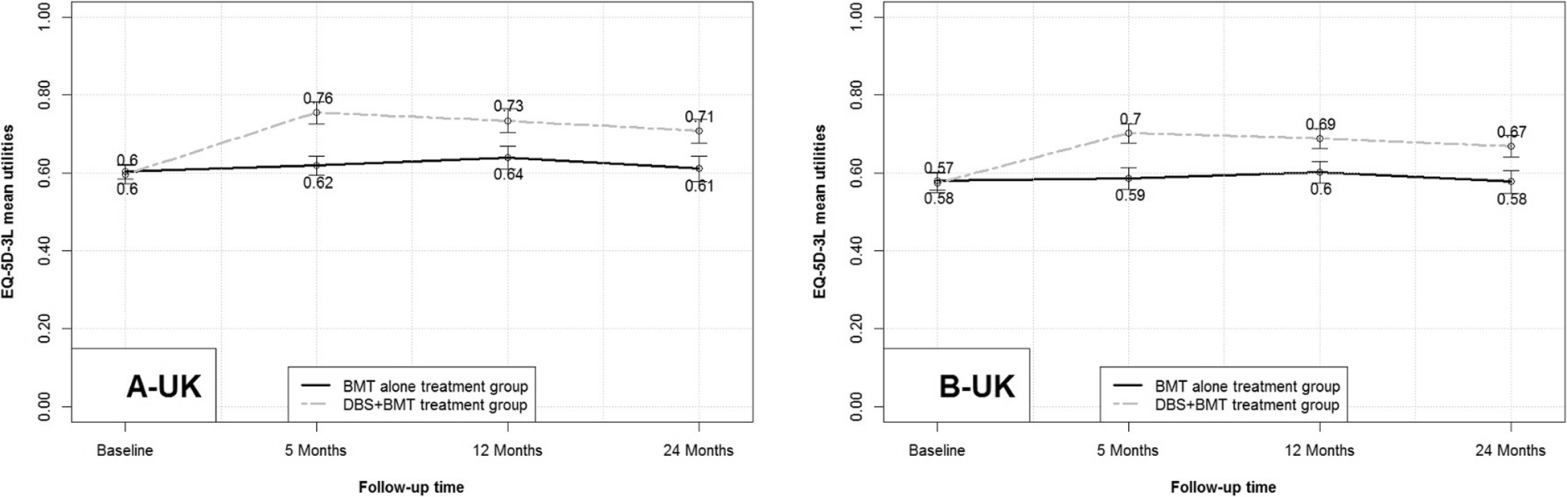
Mean UK EQ-5D-3L utilities derived using (**a**) ordinal regression and (**b**) multinomial regression

**Fig. 2:**
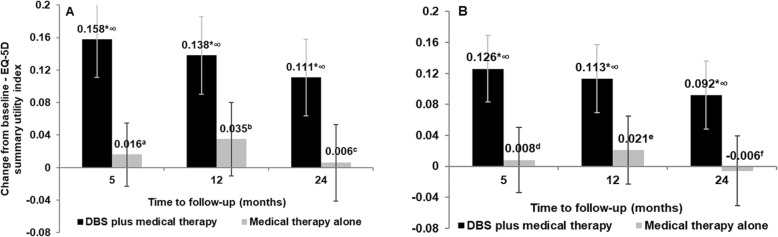
Change from baseline in mean EQ-5D-3L UK utilities derived using (**a**) ordinal regression, (**b**) multinomial regression

The predicted mean EQ-5D summary index utilities for the UK, derived by mapping the observed scores from the subscales of the PDQ-39 in the source data set, EARLYSTIM, using two different algorithms, are presented in Fig. 1 (A and B). With both algorithms, the results of the repeated measure analysis demonstrated significant between-treatment differences in mapped EQ-5D-3L utilities at all visits up to 24 months in favor of DBS (all, *p* < 0.001). The change from baseline with both algorithms was significant at all visits up to the final study visit at 24 months for the DBS group (all, *p* < 0.001) (Fig. 1). The change from baseline in the predicted mean utility for BMT was not significant at any time point with both the Young and Kent algorithms (Fig. 2).

The predicted mean differences in utilities between groups were larger with the Young algorithm (0.142, 0.103, 0.105 at five, 12 and 24 months, respectively) compared with the Kent et al. algorithm (0.118, 0.092, 0.098 at five, 12 and 24 months, respectively).

**Fig. 3:**
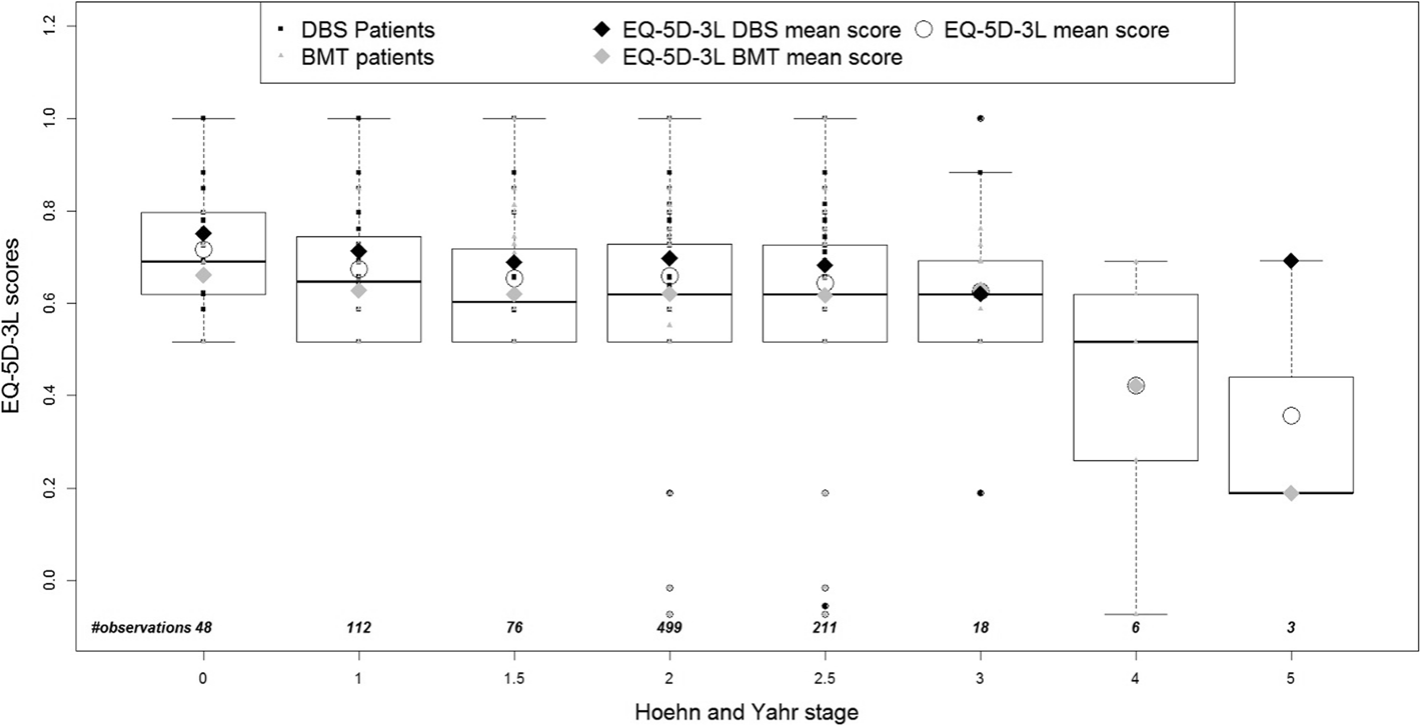
Distribution of predicted UK EQ-5D-3L utilities by PD severity - ordinal regression model

**Fig. 4:**
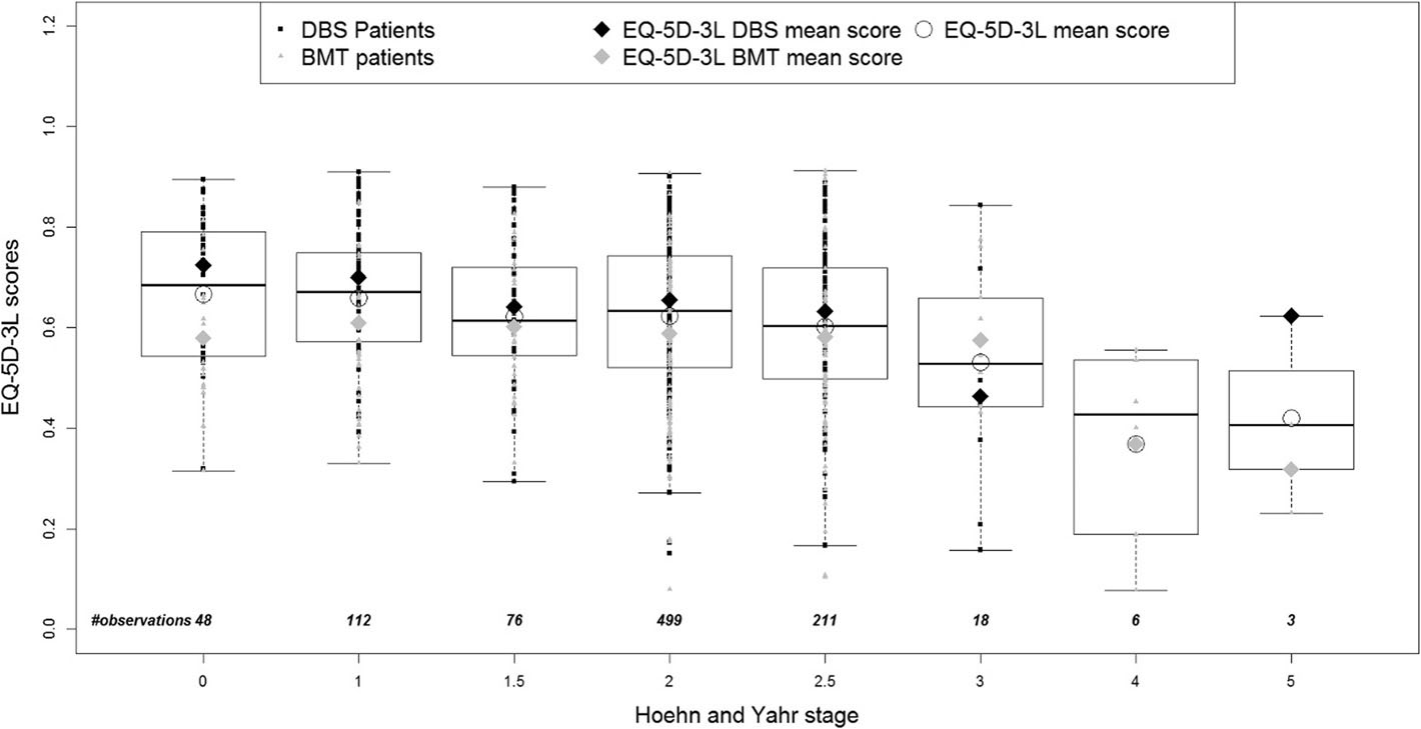
Distribution of predicted UK EQ-5D-3L utilities by PD severity - multinomial regression model

**Fig. 5:**
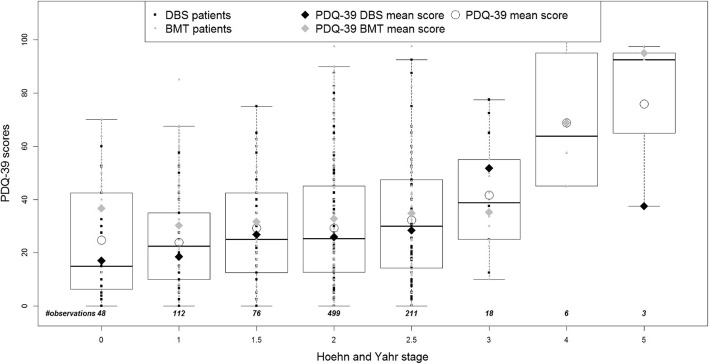
Distribution of observed patient data - PDQ-39 summary index by PD severity

Boxplots (Fig. 3 and Fig. 4) show the distribution of the predicted utilities derived using both algorithms by H&Y stage from EARLYSTIM with lower utilities corresponding to an increase in PD severity (H&Y), indicating a correlation between the two variables. This is consistent with the distribution of the mean PDQ-39 summary index by H&Y stage in EARLYSTIM (Fig. 5). However, the relationship in later H&Y stages is less reliable given fewer observations and high variability in this cohort.

The predicted mean EQ-5D summary index utilities for France are presented in Additional file 1.

## Discussion

The application of two previously developed mapping algorithms [13, 14] to patient data from the EARLYSTIM trial demonstrated a statistically significant improvement in predicted EQ-5D-3L utilities with DBS between baseline and 24 month, corroborating the improvement in the PDQ-39 summary index score, as reported at 24 months in the DBS group in the same study [20]. In contrast, there was no improvement in predicted EQ-5D-3L utilities between baseline and 24 months for the BMT group corresponding to no change in PDQ-39. Use of the mapping algorithms also revealed a statistically significant improvement in predicted EQ-5D-3L utilities with DBS compared with BMT, in line with PDQ-39 results. In contrast with the Kent algorithm [14], the Young algorithm [13] generally resulted in higher predicted utilities indicating the trend of better HRQoL. With the Young algorithm, both the change from baseline to 24 months in predicted utilities with DBS (0.111) and the difference between groups at 24 months in favor of DBS (0.105) were clinically meaningful as they exceeded the distribution of minimal clinically important difference values (0.09 to 0.10) [22].

Changes from baseline to 12 months in observed EQ-5D-3L utilities in PD patients treated with DBS plus BMT, and differences between groups, have been reported in two other studies, PD MED [21] and PD SURG [8]. In both studies, DBS resulted in a greater gain in observed utility compared with BMT (0.236 (PD MED) and 0.06 (PD SURG)). The *p*-value, only determined for the PD SURG data, showed significantly greater observed utility with DBS (*p* = 0.04). The discrepancy in utility gains between these studies may be explained by the different patient populations, as those with less advanced disease were recruited in PD MED.

Analysis of the distribution of the predicted EQ-5D-3L utilities (derived using the Kent algorithm) by the stage of PD severity (determined by H&Y stage across all EARLYSTIM visits), indicates a steady decrease in predicted utilities with disease progression at H&Y stages 1, 2 and 2.5. These data suggest that even in the early stages of PD overall HRQoL is deteriorating.

The responsiveness (ability to reflect change over time) of the predicted EQ-5D-3L utilities derived using the Young and Kent algorithms was investigated in additional analyses of the DBS and BMT groups separately, and, in order to maximize sample size, the DBS and BMT groups were combined. Statistical analyses included effect size (ES) and standardized response mean (SRM). In summary, ES and SRM utilities were larger in the DBS group than the BMT group. Moderate values were reported in the combined group. It was not possible to conclude if one algorithm was more responsive than other. An additional file shows these results in more detail (see Additional file 2).

The use of mapping to predict utilities may be considered a study limitation. While we acknowledge that mapping is less preferable to eliciting utilities directly from patients using the EQ-5D, health technology agencies have endorsed mapping as an acceptable way to derive them [5]. Best practice criteria for mapping recommends that the mapping model should be investigated using data sets that comprise the HRQoL instruments used to estimate the EQ-5D data; the statistical performance of the model must be described and its selection justified; and how the variation in mapping algorithms impacts data outputs should be explored [5]. All of these requirements have been met in the current study. Furthermore, predicting utilities using two algorithms developed using different statistical methods demonstrates a conservative approach.

This study is also limited because without empirical EQ-5D data we are unable to determine which mapping algorithm more accurately predicts utilities within the EARTLYSTIM trial. While both algorithms use different link functions, with additional covariates included in the Kent algorithm, our results are all based on regression models. However, differences between the algorithms emerged in terms of the coefficient correlates between the EQ-5D dimensions and PDQ-39 subscales. Young et al. [13] reported that the strongest positive relationships were between just one EQ-5D dimension, mobility, and the PDQ-39 subscales mobility, ADL, and bodily discomfort. In contrast, Kent et al. [14] reported strong positive associations between all EQ-5D dimensions and multiple PDQ-39 subscales including: mobility with mobility; self-care with ADL; usual activities with mobility and ADL; pain/discomfort with mobility and bodily discomfort; anxiety/depression with emotional well-being.

As the derivation of an algorithm and its application to patient data should be based on similar samples, differences in the populations included in the data sets accessed for this analysis are also important to acknowledge as a limitation. The mean age of patients recruited by Young et al. [13] and Kent et al. [14] was 65 and 71 years, respectively, in contrast to 52 years in the EARLYSTIM [20] data set used here. Also, all patients in the EARLYSTIM study had early motor complications of PD, while 76% of the Kent et al. estimation data set from PD MED were described as having “early PD” and more than 25% had progressed to H&Y stages 3, 4 or 5, subgroups that had been excluded from EARLYSTIM. In contrast, 67% of the Young et al. data set had already undergone DBS implantation, leading to the assumption that they had presented with advanced PD. While the multinomial model used by Kent et al. to derive utilities from the PD MED data set used age as a covariate, concern remains regarding the more severe PD populations in the sample, compared with the EARLYSTIM subjects.

In addition, the response pattern for disease progression cannot be interpreted reliably beyond H&Y stage 2.5 due to the small number of observations and high variability at stages 3, 4 and 5. The dearth of observations with increasingly severe PD was due to the fact that only patients between H&Y stage 0 and 2.5 on medication were recruited for EARLYSTIM. In the absence of a data set of both PDQ-39 and EQ-5D-3L observed values that would allow validation of the predicted utilities by H&Y stage, we compared the observed PDQ-39 scores and predicted EQ-5D-3L utilities with those reported by Schrag et al. [23]. We observed that the data illustrate a similar distribution of PDQ-39 for both datasets. Indeed, with highly comparable slopes, both distributions show a significant deterioration in terms of PDQ-39 with respect to the PD severity distribution. Predicted EQ-5D-3L utilities derived using the Kent algorithm showed increasing disutility as PD severity increased, albeit with a gentler slope compared with the relationship between observed EQ-5D-3L utilities and disease severity described by Schrag et al. [23].

Mapping is an innovative and promising solution to address the evidence gap made by the absence of utility data. To determine how best to fill this evidence gap health economic statisticians have developed multiple mapping models and more recently other methodologies such as bolt-on domains to utility scales [9]. While there is no consensus of opinion on which is best, mapping is likely to remain a necessary tool in the health economist’s armamentarium when it is necessary to source data from previously conducted clinical studies that lack utility data to compare treatment options for health technology assessments.

Future research is warranted to further validate the mapping models and alternative approaches, and to investigate the psychometric properties of predicted utilities in larger DBS data sets. The impact of predicted utilities mapped form both algorithms on cost-effectiveness ratios should be further explored.

## Conclusions

In the absence of the direct survey of utilities in the EARLYSTIM trial, two mapping algorithms provided acceptable alternatives for preference measurement using observed PDQ-39 scores. Analyses demonstrated a statistically significant and clinically meaningful improvement in predicted utilities with DBS compared with BMT among patients with PD and early motor complications whose disease, in spite of being at an early stage of progression, had already negatively impacted their HRQoL. Mapping utilities from disease-specific HRQoL data is an innovative statistical methodology designed to ensure that cost-effectiveness analyses can be carried out in the absence of observed utility values to facilitate decision-making so that healthcare resources can be efficiently allocated in clinical practice.

## Supplementary information

**Additional file 1.** Mean EQ-5D-3L utilities for France derived from PDQ-39 scores using the algorithms developed by (A) ordinal regression and (B) multinomial regression. EARLYSTIM trial data that were used in this analysis included the scores of the eight subscales of PDQ-39, for 127 BMT patients and 124 dB patients, measured at baseline, five, 12, and 24 month visits in addition to age and gender.

**Additional file 2.** Responsiveness of mapped utilities: comparisons between the Kent and Young algorithms. In general, the Young et al. algorithm resulted in higher predicted utilities indicating the trend of better HRQoL compared with the Kent et al. algorithm. In addition, application of the French tariffs resulted in lower predicted utility values than the UK tariffs.

## Data Availability

The data that support the findings of this study were used under license for the current study, and so are not publicly available.

## Abbreviations

BMT: Best medical therapy
DBS: Deep brain stimulation
EARLYSTIM: Controlled Trial of Deep Brain Stimulation in Early Patients with Parkinson’s Disease
EQ-5D: Euroqol 5 dimensions
EQ-5D-3L: EuroQol 5 dimensions with 3 levels of response
HRQoL: Health-related quality of life
HY: Hoehn & Yahr stage
ITT: Intention-to-treat
PD MED: Long-term effectiveness of dopamine agonists and monoamine oxidase B inhibitors compared with levodopa as initial treatment for Parkinson’s disease
PD SURG: Deep brain stimulation plus best medical therapy versus best medical therapy alone for advanced Parkinson’s disease
PD: Parkinson’s disease
PDQ-39: 39-item Parkinson’s disease questionnaire eight dimensions
PDQ-8: 8-item Parkinson’s disease questionnaire eight dimensions
