# Supplementary figures and images for: Parkinson’s disease with early motor complications: predicting EQ-5D- 3L utilities from PDQ-39 data in the EARLYSTIM trial
Source: Health Qual Life Outcomes. 2020 Mar 2;18:49. doi: 10.1186/s12955-020-01299-y (PMC7053067; doi:10.1186/s12955-020-01299-y)

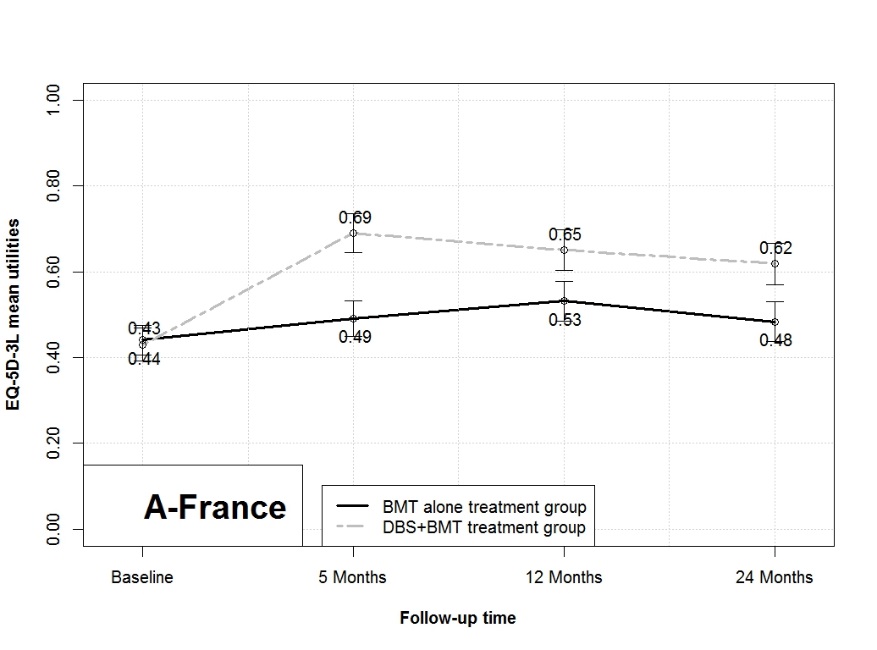

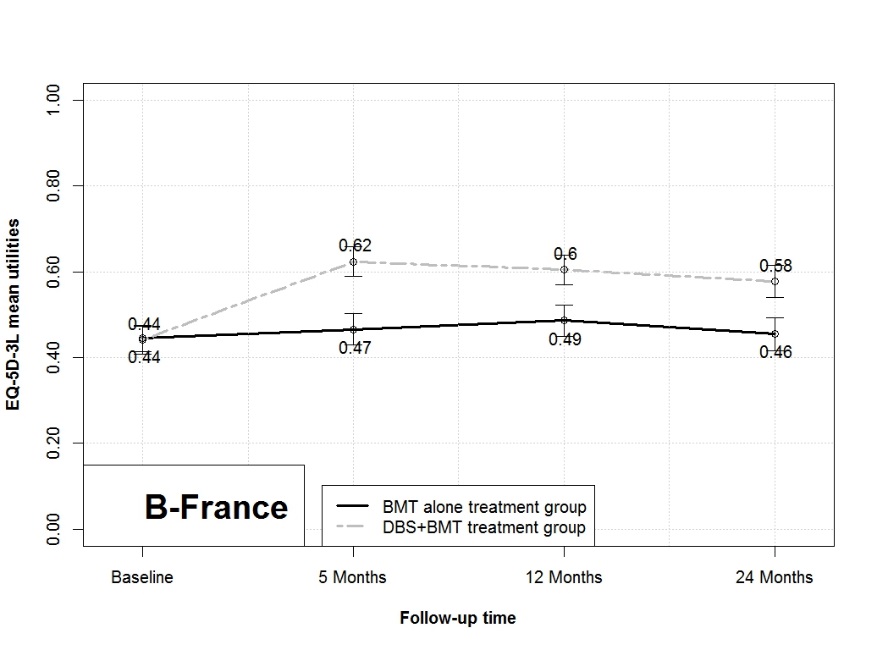

Supplement: Supplementary file 1 — Additional file 1. Mean EQ-5D-3L utilities for France derived from PDQ-39 scores using the algorithms developed by (A) ordinal regression and (B) multinomial regression. EARLYSTIM trial data that were used in this analysis included the scores of the eight subscales of PDQ-39, for 127 BMT patients and 124 dB patients, measured at baseline, five, 12, and 24 month visits in addition to age and gender. [file 12955_2020_1299_MOESM1_ESM.docx]
