# Supplementary material for: Parkinson’s disease with early motor complications: predicting EQ-5D- 3L utilities from PDQ-39 data in the EARLYSTIM trial
Source: Health Qual Life Outcomes. 2020 Mar 2;18:49. doi: 10.1186/s12955-020-01299-y (PMC7053067; doi:10.1186/s12955-020-01299-y)
Supplement: Supplementary file 2 — Additional file 2. Responsiveness of mapped utilities: comparisons between the Kent and Young algorithms. In general, the Young et al. algorithm resulted in higher predicted utilities indicating the trend of better HRQoL compared with the Kent et al. algorithm. In addition, application of the French tariffs resulted in lower predicted utility values than the UK tariffs. [file 12955_2020_1299_MOESM2_ESM.doc]

# Responsiveness of mapped utilities: comparisons between the Kent and Young algorithms

# Methods

Responsiveness is the ability of an instrument to reflect change in the health of a population over time and can be seen as a form of validity. As recommended by Brazier *et al.* [1], responsiveness is usually assessed statistically using measures such as the effect size (ES) and the Standardized Response Mean (SRM).

The responsiveness analysis reported here assessed the sensitivity to change of the mapped EQ-5D-3 L values from both the Young [2] and Kent [3] algorithms. This included an analysis of the magnitude of the change in score between two study time points. Change was assessed before and after an intervention, at 5, 12 and 24 months. The magnitude of the change reflected in the measures between the time points was assessed using the SRM statistic, which is calculated by dividing the mean change on the measure by the SD of the change, and the ES, calculated by dividing the mean change in score by the SD at baseline. Within this review, the following Cohen’s [4] categories for magnitude of effect size were used: ≥ 0.80 = large, < 0.80 and ≥ 0.50 =moderate, and 0.30 to < 0.50 = small. However, these categories need to be used with some care as there is no gold standard and their application sometimes uses indirect indicators of the concept (e.g., symptoms rather than health-related quality of life (HRQoL)).

# Results

Table 1 shows the responsiveness statistics at three follow-up visits for both the mapped EQ-5D-3 L measurements and the original PDQ-39 scores, considering only observations of subjects in the deep brain stimulation (DBS) group in EARLYSTIM. Young EQ-5D-3 L mapped values give the largest effect size at the three follow-up visits while Kent EQ-5D mapped measures show the highest SRM. Overall, for both effect size and SRM, as responsiveness of the three outcomes was very similar, there is no evidence that one algorithm was more responsive than the other.

Table 2 shows the same responsiveness statistics for the control group, patients who received best medical therapy (BMT) in EARLYSTIM. All responsiveness statistics were small, with a small advantage for the Young outcomes. The fact that responsiveness is very small can be explained by the minor change in HRQoL scores for the control group within this dataset.

Table 1 Responsiveness of PDQ-39 scores and mapped EQ-5D-3 L utilities to longitudinal change in the DBS cohort

|  | **Effect Size** | | | **Standardized Response Mean** | | |
| --- | --- | --- | --- | --- | --- | --- |
| **Visit (months)** | PDQ-39_DBS | Predicted utilities | | PDQ-39_DBS | Predicted utilities | |
|  |  | Kent_DBS | Young_DBS |  | Kent_DBS | Young_DBS |
| **5**  (n=119) | -0.85 | 0.9 | **1.17** | -0.93 | **0.94** | 0.88 |
| **12**  (n=120) | -0.74 | 0.81 | **1.02** | -0.75 | **0.81** | 0.75 |
| **24**  (n=120) | -0.59 | 0.66 | **0.82** | -0.56 | **0.68** | 0.62 |

Change was assessed from baseline through the five, 12, and 24 months visits. Highest (absolute) values are shown in bold font to indicate the most responsive measure. DBS = Deep brain stimulation; Kent = Application of the Kent algorithm [3] to the DBS cohort; Young = Application of the Young algorithm [2] to the DBS cohort.

Table 2 Responsiveness of PDQ-39 scores and mapped EQ-5D-3 L utilities to longitudinal change in the BMT cohort

|  | **Effect Size** | | | **Standardized Response Mean** | | |
| --- | --- | --- | --- | --- | --- | --- |
| **Visit (months)** | PDQ-39_BMT | Predicted utilities | | PDQ-39_BMT | Predicted utilities | |
|  |  | Kent_BMT | Young_BMT |  | Kent_BMT | Young_BMT |
| **5**  (n=125) | -0.1 | 0.06 | **0.15** | -0.1 | 0.07 | **0.13** |
| **12**  (n=125) | -0.12 | 0.16 | **0.32** | -0.13 | 0.19 | **0.24** |
| **24**  (n=123) | 0.02 | -0.04 | **0.05** | 0.02 | **-0.04** | **0.04** |

Change was assessed from baseline through the five, 12, and 24 months visits. Highest (absolute) values are shown in bold font to indicate the most responsive measure. BMT = Best medical therapy; Kent = Application of the Kent algorithm [3] to the BMT cohort; Young = Application of the Young algorithm [2] to the BMT cohort.

As the objective of this supplementary analysis was to explore responsiveness in terms of the ability to capture change over time, rather than to compare responsiveness between groups, an alternative method is to maximize the sample size by combining the observations in both treatment groups. Hence, Table 3 shows that, for both the first two follow-up visits, the effect size and the SMRs are in the moderate range. Young’s EQ-5D-3 L mapped utilities show the highest responsiveness values but the difference between the three outcomes is very small. Again, with these analyses we cannot conclude if one algorithm is more responsive than other.

Table 3 Responsiveness of PDQ-39 scores and mapped EQ-5D-3 L utilities to longitudinal change in pooled DBS and BMT observations

|  | **Effect Size** | | | **Standardized Response Mean** | | |
| --- | --- | --- | --- | --- | --- | --- |
| **Visit (months)** | PDQ-39 | Predicted utilities | | PDQ-39 | Predicted utilities | |
|  |  | Kent | Young |  | Kent | Young |
| **5**  (n=244) | -0.48 | 0.48 | **0.5** | -0.48 | 0.48 | **0.5** |
| **12**  (n=245) | -0.43 | 0.48 | **0.5** | -0.43 | **0.49** | **0.49** |
| **24**  (n=243) | -0.29 | 0.31 | **0.34** | -0.28 | 0.3 | **0.32** |

Change was assessed from baseline through the five, 12, and 24 months visits. Highest (absolute) values are shown in bold font to indicate the most responsive measure. DBS = Deep brain stimulation; BMT = Best medical therapy; Kent = Application of the Kent algorithm [3]; Young = Application of the Young algorithm [2].

# Conclusions

Both algorithms showed a large responsiveness for DBS patients, small responsiveness for BMT patients and moderate values when both treatment groups were combined. This is mainly due to the higher disease specific HRQoL gain observed from the change in PDQ-39 for DBS compared with BMT patients. Regarding the comparison between both algorithms, differences in responsiveness were neither significant nor consistent between both statistical measures, i.e., ES and SRM. To conclude, we cannot state that one algorithm is more responsive than the other. Further testing of responsiveness and other psychometric properties of these mapped generic measures could be undertaken using a larger data set.

# References

1. Brazier J, Connell J, Papaioannou D, Mukuria C, Mulhern B, Peasgood T, et al. A systematic review, psychometric analysis and qualitative assessment of generic preference-based measures of health in mental health populations and the estimation of mapping functions from widely used specific measures. Health Technol Assess. 2014;18:1-188.
2. Young M, Ng SK, Mellick G, Scuffham P. Mapping of the PDQ-39 to EQ-5D scores in patients with Parkinson's disease. Qual Life Res. 2013;22:1065-72.
3. Kent S, Gray A, Schlackow I, Jenkinson C, McIntosh E. Mapping from the Parkinson's Disease Questionnaire PDQ-39 to the Generic EuroQol EQ-5D-3L: The Value of Mixture Models. Med Decis Making. 2015;35:902-11.
4. Cohen J. Statistical Power Analysis for the Behavioural Sciences. 2nd ed. Hillsdale, New Jersey: Lawrence Erlbaum Associates; 1988.
